# (8-Benzoyl-2,7-dieth­oxy­naphthalen-1-yl)(phen­yl)methanone

**DOI:** 10.1107/S1600536812049963

**Published:** 2012-12-12

**Authors:** Atsumi Isogai, Takehiro Tsumuki, Shun Murohashi, Akiko Okamoto, Noriyuki Yonezawa

**Affiliations:** a Department of Organic and Polymer Materials Chemistry, Tokyo University of Agriculture & Technology, 2-24-16 Naka-machi, Koganei, Tokyo 184-8588, Japan

## Abstract

In the title compound, C_28_H_24_O_4_, the benzoyl groups at the 1- and 8-positions of the naphthalene ring system are aligned almost anti­parallel, and the benzene rings make a dihedral angle of 20.03 (7)°. The dihedral angles between the benzene rings and the naphthalene ring system are 68.42 (5) and 71.69 (5)°. In the crystal, adjacent mol­ecules are linked *via* C—H⋯O hydrogen bonds, forming chains propagating along [100].

## Related literature

For electrophilic aroylation of naphthalene derivatives, see: Okamoto & Yonezawa (2009 ▶); Okamoto *et al.* (2011 ▶). For the structures of closely related compounds, see: Nakaema *et al.* (2008 ▶); Nishijima *et al.* (2010 ▶); Sasagawa *et al.* (2011 ▶); Tsumuki *et al.* (2011 ▶); Muto *et al.* (2012 ▶).
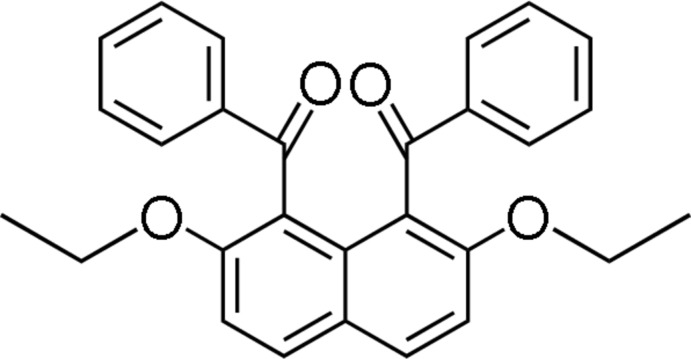


## Experimental

### 

#### Crystal data

C_28_H_24_O_4_
*M* *_r_* = 424.47
Monoclinic, 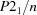

*a* = 7.92185 (14) Å
*b* = 20.6794 (4) Å
*c* = 14.2130 (3) Å
β = 106.043 (1)°
*V* = 2237.68 (7) Å^3^
*Z* = 4
Cu *K*α radiation
μ = 0.67 mm^−1^
*T* = 193 K
0.60 × 0.50 × 0.10 mm

#### Data collection

Rigaku R-AXIS RAPID diffractometer
Absorption correction: numerical (*NUMABS*; Higashi, 1999 ▶) *T*_min_ = 0.689, *T*_max_ = 0.936
39782 measured reflections
4076 independent reflections
3736 reflections with *I* > 2σ(*I*)
*R*_int_ = 0.041

#### Refinement

*R*[*F* ^2^ > 2σ(*F* ^2^)] = 0.035
*wR*(*F* ^2^) = 0.091
*S* = 1.05
4076 reflections
292 parameters
H-atom parameters constrained
Δρ_max_ = 0.23 e Å^−3^
Δρ_min_ = −0.16 e Å^−3^

### 

Data collection: *PROCESS-AUTO* (Rigaku, 1998 ▶); cell refinement: *PROCESS-AUTO*; data reduction: *PROCESS-AUTO*; program(s) used to solve structure: *IL MILIONE* (Burla *et al.*, 2007 ▶); program(s) used to refine structure: *SHELXL97* (Sheldrick, 2008 ▶); molecular graphics: *ORTEPIII* (Burnett & Johnson, 1996 ▶); software used to prepare material for publication: *SHELXL97*.

## Supplementary Material

Crystal structure: contains datablock(s) I, global. DOI: 10.1107/S1600536812049963/su2538sup1.cif

Structure factors: contains datablock(s) I. DOI: 10.1107/S1600536812049963/su2538Isup2.hkl

Supplementary material file. DOI: 10.1107/S1600536812049963/su2538Isup3.cml

Additional supplementary materials:  crystallographic information; 3D view; checkCIF report

## Figures and Tables

**Table 1:** Hydrogen-bond geometry (Å, °)

*D*—H⋯*A*	*D*—H	H⋯*A*	*D*⋯*A*	*D*—H⋯*A*
C14—H14⋯O3^i^	0.95	2.37	3.2404 (16)	153
C21—H21⋯O4^ii^	0.95	2.39	3.3326 (16)	171

Symmetry codes: (i) 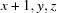; (ii) 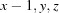.

**Fig. 1.**
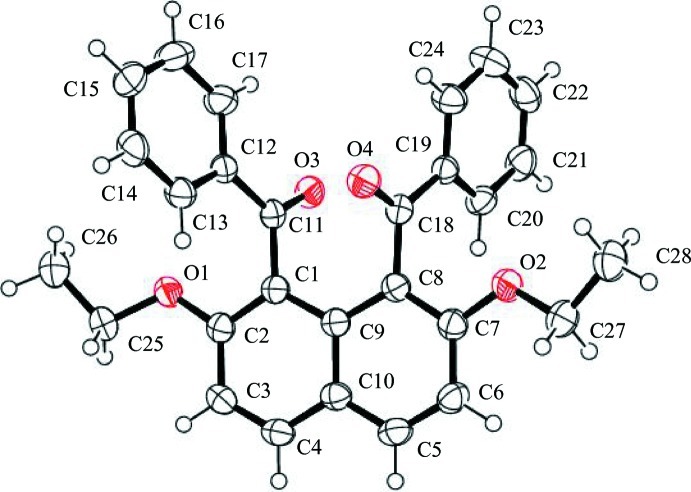
Molecular structure of the title molecule, with atom numbering. Displacement ellipsoids are drawn at the 50% probability level.

**Fig. 2.**
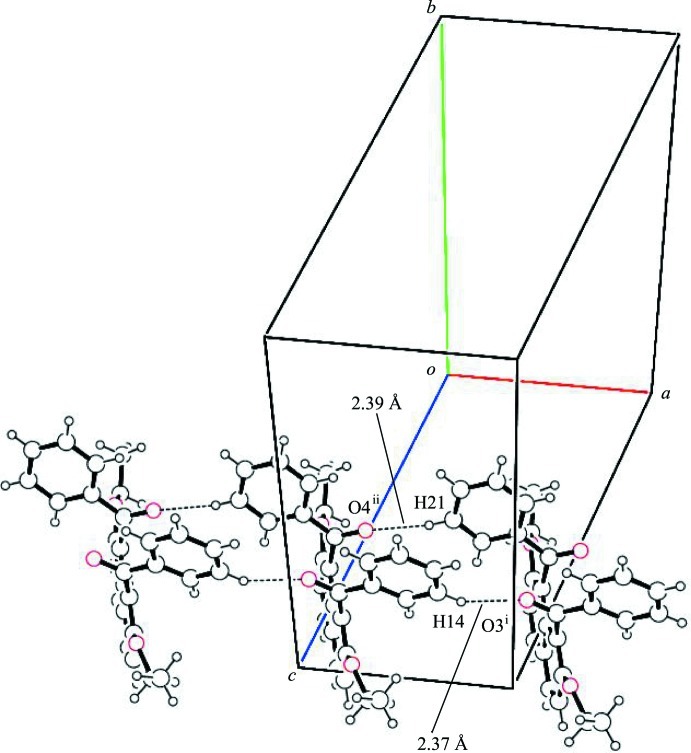
A partial view of the crystal packing of the title compound, showing the intermolecular C-H···O hydrogen bonds (see Table 1 for details; symmetry codes: (i) x+1, y, z; (ii) x-1, y, z).

## supplementary crystallographic information

### Comment

In the course of our study on selective electrophilic aromatic aroylation of naphthalene ring core, 1,8-diaroylnaphthalene compounds have proved to be formed regioselectively by the aid of a suitable acidic mediator (Okamoto & Yonezawa, 2009, Okamoto *et al.*, 2011). Recently, we have reported the X-ray crystal structure of 1,8-diaroyled 2,7-dimethoxynaphthalene derivatives such as 1,8-bis(4-aminobenzoyl)-2,7-dimethoxynaphthalene (Nishijima *et al.*, 2010), [8-(4-butoxybenzoyl)-2,7-dimethoxynaphthalen-1-yl](4-butoxyphenyl)methanone [1,8-bis(4-butoxybenzoyl)-2,7-dimethoxynaphthalene] (Sasagawa *et al.*, 2011), [2,7-dimethoxy-8-(2-naphthoyl)naphthalene-1-yl](naphthalene-2-yl)methanone [2,7-dimethoxy-1,8-bis(2-naphthoyl)naphthalene] (Tsumuki *et al.*, 2011), and (3,5-dimethylphenyl)[8-(3,5-dimethylbenzoyl)-2,7-dimethoxynaphthalen-1-yl]methanone (Muto *et al.*, 2012). The simplest molecule in these analogues, 1,8-dibenzoyl-2,7-dimethoxynaphthalene (Nakaema *et al.*, 2008), lies across a crystallographic 2-fold axis and the molecular packing is stabilized by C—H···O interactions between carbonyl groups and benzene ring and π···π interactions between benzene rings. As a part of our ongoing studies on the molecular structures of these kinds of homologous molecules, the X-ray crystal structure of the title compound, is reported on herein.

The molecule structure of the title molecule is illustrated in Fig. 1. The two benzoyl groups are situated in an (*anti*)-orientation and twisted away from the attached naphthalene ring. The dihedral angle between the planes of the two benzene rings (C12-C17 & C19-C24) is 20.03 (7)°. The dihedral angles between these benzene rings and the naphthalene (C1-C10) ring are 68.42 (5)° and 71.69 (5)°. These dihedral angles are similar to that reported for 1,8-dibenzoyl-2,7-dimethoxynaphthalene [80.25 (6)°; Nakaema *et al.*, 2008]. The torsion angles between the carbonyl groups and the naphthalene ring are -63.28 (14)° [C9—C1—C11—O3] and -66.19 (14)° [C9—C8—C18—O4]. The C═O groups lie almost in the plane of the attached benzene ring with torsion angles equal to 1.58 (17)° [O3—C11—C12—C17] and 1.44 (17)° [O4—C18—C19—C24].

In the crystal, the molecular packing of the title compound is mainly stabilized by two types of C—H···O interactions involving adjacent molecules and leading to the formation of chains along the *a* axis (Table 1 and Fig. 2).

### Experimental

To a 100 ml flask, benzoyl chloride (422 mg, 3.0 mmol), titanium chloride (1.71 g, 9.0 mmol) and methylene chloride (1.5 ml) were stirred at 298 K under nitrogen atmosphere. To reaction mixture thus obtained, 2,7-diethoxynaphtharene (216 mg, 1.0 mmol) and methylene chloride (1.0 ml) were added. After the reaction mixture was stirred at r.t. for 24 h, it was poured into ice-cold water (20 ml). The aqueous layer was extracted with CHCl_3_ (10 ml × 3). The combined extracts were washed with 2 *M* aqueous NaOH followed by washing with brine. The organic layers thus obtained were dried over anhydrous MgSO_4_. The solvent was removed under reduced pressure to give cake. The crude product was purified by recrystallization from CHCl_3_/methanol (1: 2 *v*/*v*) solution (81% yield). Colorless platelet single crystals suitable for X-ray diffraction were obtained by repeated crystallization from a CHCl_3_/ methanol (1:2 *v*/*v*) solution (52% yield). Anal. Calcd for C_28_H_24_O_4_: C 79.22, H5.70. Found: C 79.32, H 5.84.; M.p. = 467–468 K. Spectroscopic data for the title compound are available in the archived CIF.

### Refinement

All the H atoms were located in a difference Fourier map and were subsequently refined as riding atoms: C—H = 0.95 (aromatic), 0.98 (methyl) and 0.99 (methylene) Å, with *U*_iso_(H) = 1.2 *U*_eq_(C).

### Crystal data

**Table:**

C_28_H_24_O_4_	*F*(000) = 896
*M**_r_* = 424.47	*D*_x_ = 1.260 Mg m^−^^3^
Monoclinic, *P*2_1_/*n*	Cu *K*α radiation, λ = 1.54187 Å
Hall symbol: -P 2yn	Cell parameters from 37233 reflections
*a* = 7.92185 (14) Å	θ = 3.2–68.3°
*b* = 20.6794 (4) Å	µ = 0.67 mm^−^^1^
*c* = 14.2130 (3) Å	*T* = 193 K
β = 106.043 (1)°	Needle, colourless
*V* = 2237.68 (7) Å^3^	0.60 × 0.50 × 0.10 mm
*Z* = 4	

### Data collection

**Table:**

Rigaku R-AXIS RAPID diffractometer	4076 independent reflections
Radiation source: fine-focus sealed tube	3736 reflections with *I* > 2σ(*I*)
Graphite monochromator	*R*_int_ = 0.041
Detector resolution: 10.000 pixels mm^-1^	θ_max_ = 68.2°, θ_min_ = 3.9°
ω scans	*h* = −9→9
Absorption correction: numerical (*NUMABS*; Higashi, 1999)	*k* = −24→24
*T*_min_ = 0.689, *T*_max_ = 0.936	*l* = −17→17
39782 measured reflections	

### Refinement

**Table:**

Refinement on *F*^2^	Secondary atom site location: difference Fourier map
Least-squares matrix: full	Hydrogen site location: inferred from neighbouring sites
*R*[*F*^2^ > 2σ(*F*^2^)] = 0.035	H-atom parameters constrained
*wR*(*F*^2^) = 0.091	*w* = 1/[σ^2^(*F*_o_^2^) + (0.0439*P*)^2^ + 0.5163*P*] where *P* = (*F*_o_^2^ + 2*F*_c_^2^)/3
*S* = 1.05	(Δ/σ)_max_ < 0.001
4076 reflections	Δρ_max_ = 0.23 e Å^−^^3^
292 parameters	Δρ_min_ = −0.16 e Å^−^^3^
0 restraints	Extinction correction: *SHELXL97* (Sheldrick, 2008), Fc^*^=kFc[1+0.001xFc^2^λ^3^/sin(2θ)]^-1/4^
Primary atom site location: structure-invariant direct methods	Extinction coefficient: 0.0075 (3)

### Special details

**Table:**

Experimental. Spectroscopic data for the title compound: ^1^H NMR δ (300 MHz, CDCl_3_); 0.91 (6*H*, t, *J* = 6.9 Hz), 3.95 (4*H*, q, *J* = 6.9 Hz), 7.16 (2*H*, d, *J* = 9.0 Hz), 7.35 (4*H*, t, *J* = 7.2 Hz), 7.49(2*H*, t, *J* = 7.2 Hz), 7.73 (4*H*, d, *J* = 7.2 Hz), 7.91 (2*H*, d, *J* = 9.0 Hz) p.p.m.; ^13^C NMR δ (75 MHz, CDCl_3_); 13.92, 64.42, 111.89, 121.27, 125.05, 127.43, 128.54, 129.80, 131.79, 131.95, 138.93, 155.52, 197.04 p.p.m.; IR (KBr, cm^-1^): 1665 (C═ O), 1612, 1510, 1452 (Ar), 1266 (═C—O—C).
Geometry. All e.s.d.'s (except the e.s.d. in the dihedral angle between two l.s. planes) are estimated using the full covariance matrix. The cell e.s.d.'s are taken into account individually in the estimation of e.s.d.'s in distances, angles and torsion angles; correlations between e.s.d.'s in cell parameters are only used when they are defined by crystal symmetry. An approximate (isotropic) treatment of cell e.s.d.'s is used for estimating e.s.d.'s involving l.s. planes.
Refinement. Refinement of *F*^2^ against ALL reflections. The weighted *R*-factor *wR* and goodness of fit *S* are based on *F*^2^, conventional *R*-factors *R* are based on *F*, with *F* set to zero for negative *F*^2^. The threshold expression of *F*^2^ > σ(*F*^2^) is used only for calculating *R*-factors(gt) *etc*. and is not relevant to the choice of reflections for refinement. *R*-factors based on *F*^2^ are statistically about twice as large as those based on *F*, and *R*- factors based on ALL data will be even larger.

### Fractional atomic coordinates and isotropic or equivalent isotropic displacement parameters (Å^2^)

**Table:**

	*x*	*y*	*z*	*U*_iso_*/*U*_eq_	
O1	0.29349 (11)	0.08669 (4)	1.04839 (6)	0.0381 (2)	
O2	−0.21634 (12)	0.01858 (4)	0.55669 (6)	0.0458 (2)	
O3	−0.02728 (10)	0.16304 (4)	0.86895 (6)	0.0383 (2)	
O4	0.08625 (10)	0.12983 (4)	0.66958 (6)	0.0414 (2)	
C1	0.12835 (13)	0.06508 (5)	0.88895 (8)	0.0299 (2)	
C2	0.21642 (14)	0.04134 (5)	0.98017 (8)	0.0325 (3)	
C3	0.21668 (15)	−0.02515 (6)	1.00229 (9)	0.0367 (3)	
H3	0.2781	−0.0406	1.0654	0.044*	
C4	0.12768 (15)	−0.06697 (6)	0.93199 (9)	0.0375 (3)	
H4	0.1268	−0.1117	0.9471	0.045*	
C5	−0.05370 (16)	−0.08988 (6)	0.76527 (10)	0.0398 (3)	
H5	−0.0536	−0.1344	0.7817	0.048*	
C6	−0.14091 (16)	−0.07056 (6)	0.67303 (9)	0.0410 (3)	
H6	−0.2012	−0.1012	0.6258	0.049*	
C7	−0.14038 (15)	−0.00448 (6)	0.64861 (9)	0.0361 (3)	
C8	−0.05794 (14)	0.04132 (5)	0.71690 (8)	0.0314 (2)	
C9	0.03474 (13)	0.02192 (5)	0.81381 (8)	0.0302 (2)	
C10	0.03651 (14)	−0.04555 (5)	0.83711 (9)	0.0341 (3)	
C11	0.11515 (14)	0.13756 (5)	0.87770 (8)	0.0299 (2)	
C12	0.27340 (14)	0.17695 (5)	0.87977 (8)	0.0320 (2)	
C13	0.43562 (15)	0.14857 (6)	0.88777 (9)	0.0390 (3)	
H13	0.4468	0.1028	0.8910	0.047*	
C14	0.58108 (16)	0.18650 (7)	0.89110 (10)	0.0477 (3)	
H14	0.6918	0.1668	0.8966	0.057*	
C15	0.56497 (18)	0.25260 (7)	0.88645 (11)	0.0542 (4)	
H15	0.6650	0.2787	0.8893	0.065*	
C16	0.4039 (2)	0.28145 (7)	0.87758 (13)	0.0586 (4)	
H16	0.3933	0.3272	0.8739	0.070*	
C17	0.25816 (17)	0.24361 (6)	0.87402 (11)	0.0455 (3)	
H17	0.1474	0.2634	0.8676	0.055*	
C18	−0.05219 (14)	0.10942 (5)	0.67903 (8)	0.0310 (2)	
C19	−0.21431 (14)	0.14952 (5)	0.65338 (8)	0.0310 (2)	
C20	−0.37066 (15)	0.12789 (6)	0.66818 (9)	0.0390 (3)	
H20	−0.3762	0.0860	0.6947	0.047*	
C21	−0.51890 (16)	0.16678 (7)	0.64465 (10)	0.0459 (3)	
H21	−0.6257	0.1516	0.6547	0.055*	
C22	−0.51078 (18)	0.22760 (7)	0.60655 (10)	0.0483 (3)	
H22	−0.6126	0.2541	0.5898	0.058*	
C23	−0.35529 (19)	0.25015 (7)	0.59266 (11)	0.0531 (4)	
H23	−0.3498	0.2924	0.5673	0.064*	
C24	−0.20769 (17)	0.21117 (6)	0.61576 (10)	0.0435 (3)	
H24	−0.1010	0.2267	0.6058	0.052*	
C25	0.42914 (16)	0.06661 (6)	1.13328 (9)	0.0420 (3)	
H25A	0.5139	0.0379	1.1140	0.050*	
H25B	0.3777	0.0429	1.1792	0.050*	
C26	0.5191 (2)	0.12683 (7)	1.18056 (11)	0.0571 (4)	
H26A	0.4357	0.1534	1.2032	0.068*	
H26B	0.5624	0.1513	1.1329	0.068*	
H26C	0.6181	0.1151	1.2365	0.068*	
C27	−0.26626 (18)	−0.02602 (7)	0.47696 (9)	0.0469 (3)	
H27A	−0.3661	−0.0530	0.4830	0.056*	
H27B	−0.1667	−0.0549	0.4766	0.056*	
C28	−0.31808 (19)	0.01303 (7)	0.38490 (10)	0.0515 (3)	
H28A	−0.2205	0.0413	0.3817	0.062*	
H28B	−0.4211	0.0394	0.3844	0.062*	
H28C	−0.3465	−0.0160	0.3282	0.062*	

### Atomic displacement parameters (Å^2^)

**Table:**

	*U*^11^	*U*^22^	*U*^33^	*U*^12^	*U*^13^	*U*^23^
O1	0.0391 (4)	0.0380 (4)	0.0334 (4)	0.0027 (3)	0.0035 (3)	0.0007 (3)
O2	0.0568 (6)	0.0368 (5)	0.0362 (5)	0.0032 (4)	−0.0001 (4)	−0.0053 (4)
O3	0.0266 (4)	0.0375 (4)	0.0499 (5)	0.0040 (3)	0.0094 (4)	−0.0037 (4)
O4	0.0306 (4)	0.0456 (5)	0.0502 (5)	−0.0024 (3)	0.0149 (4)	0.0054 (4)
C1	0.0243 (5)	0.0317 (6)	0.0356 (6)	0.0007 (4)	0.0114 (4)	0.0011 (4)
C2	0.0275 (5)	0.0355 (6)	0.0355 (6)	0.0016 (4)	0.0105 (5)	−0.0005 (5)
C3	0.0343 (6)	0.0389 (6)	0.0372 (6)	0.0056 (5)	0.0103 (5)	0.0077 (5)
C4	0.0375 (6)	0.0304 (6)	0.0463 (7)	0.0036 (5)	0.0146 (5)	0.0069 (5)
C5	0.0424 (7)	0.0278 (6)	0.0502 (7)	0.0005 (5)	0.0146 (6)	−0.0009 (5)
C6	0.0421 (7)	0.0326 (6)	0.0463 (7)	−0.0014 (5)	0.0090 (6)	−0.0082 (5)
C7	0.0335 (6)	0.0362 (6)	0.0376 (6)	0.0029 (5)	0.0081 (5)	−0.0026 (5)
C8	0.0271 (5)	0.0310 (6)	0.0371 (6)	0.0027 (4)	0.0104 (5)	−0.0006 (5)
C9	0.0252 (5)	0.0309 (5)	0.0366 (6)	0.0017 (4)	0.0120 (4)	0.0006 (4)
C10	0.0314 (6)	0.0305 (6)	0.0423 (7)	0.0022 (4)	0.0136 (5)	0.0014 (5)
C11	0.0263 (5)	0.0336 (6)	0.0288 (5)	0.0019 (4)	0.0059 (4)	−0.0014 (4)
C12	0.0286 (6)	0.0341 (6)	0.0316 (6)	−0.0007 (4)	0.0054 (4)	0.0023 (4)
C13	0.0304 (6)	0.0393 (6)	0.0472 (7)	0.0021 (5)	0.0104 (5)	0.0066 (5)
C14	0.0273 (6)	0.0594 (8)	0.0551 (8)	−0.0003 (5)	0.0090 (6)	0.0139 (7)
C15	0.0394 (7)	0.0570 (9)	0.0628 (9)	−0.0168 (6)	0.0085 (6)	0.0119 (7)
C16	0.0539 (8)	0.0367 (7)	0.0840 (11)	−0.0087 (6)	0.0171 (8)	0.0089 (7)
C17	0.0377 (7)	0.0362 (7)	0.0616 (8)	0.0017 (5)	0.0119 (6)	0.0057 (6)
C18	0.0291 (5)	0.0345 (6)	0.0291 (6)	−0.0020 (4)	0.0077 (4)	−0.0019 (4)
C19	0.0307 (6)	0.0317 (6)	0.0295 (6)	−0.0002 (4)	0.0065 (4)	−0.0018 (4)
C20	0.0327 (6)	0.0339 (6)	0.0501 (7)	−0.0020 (5)	0.0110 (5)	−0.0004 (5)
C21	0.0296 (6)	0.0488 (7)	0.0579 (8)	0.0000 (5)	0.0097 (6)	−0.0075 (6)
C22	0.0419 (7)	0.0497 (8)	0.0476 (8)	0.0163 (6)	0.0030 (6)	−0.0005 (6)
C23	0.0581 (8)	0.0433 (7)	0.0585 (9)	0.0130 (6)	0.0173 (7)	0.0170 (6)
C24	0.0419 (7)	0.0410 (7)	0.0498 (8)	0.0018 (5)	0.0162 (6)	0.0098 (6)
C25	0.0373 (6)	0.0481 (7)	0.0360 (6)	0.0040 (5)	0.0026 (5)	0.0033 (5)
C26	0.0538 (8)	0.0551 (9)	0.0504 (8)	−0.0017 (7)	−0.0055 (7)	−0.0013 (7)
C27	0.0516 (8)	0.0469 (7)	0.0394 (7)	−0.0044 (6)	0.0082 (6)	−0.0101 (6)
C28	0.0513 (8)	0.0602 (9)	0.0406 (7)	−0.0046 (6)	0.0091 (6)	−0.0049 (6)

### Geometric parameters (Å, º)

**Table:**

O1—C2	1.3657 (14)	C15—C16	1.382 (2)
O1—C25	1.4374 (14)	C15—H15	0.9500
O2—C7	1.3645 (15)	C16—C17	1.3841 (18)
O2—C27	1.4297 (15)	C16—H16	0.9500
O3—C11	1.2201 (13)	C17—H17	0.9500
O4—C18	1.2167 (13)	C18—C19	1.4870 (15)
C1—C2	1.3815 (16)	C19—C20	1.3865 (16)
C1—C9	1.4315 (15)	C19—C24	1.3890 (17)
C1—C11	1.5079 (15)	C20—C21	1.3858 (17)
C2—C3	1.4104 (16)	C20—H20	0.9500
C3—C4	1.3616 (17)	C21—C22	1.378 (2)
C3—H3	0.9500	C21—H21	0.9500
C4—C10	1.4135 (17)	C22—C23	1.381 (2)
C4—H4	0.9500	C22—H22	0.9500
C5—C6	1.3620 (18)	C23—C24	1.3830 (18)
C5—C10	1.4108 (17)	C23—H23	0.9500
C5—H5	0.9500	C24—H24	0.9500
C6—C7	1.4101 (17)	C25—C26	1.4985 (19)
C6—H6	0.9500	C25—H25A	0.9900
C7—C8	1.3839 (16)	C25—H25B	0.9900
C8—C9	1.4277 (16)	C26—H26A	0.9800
C8—C18	1.5129 (15)	C26—H26B	0.9800
C9—C10	1.4331 (15)	C26—H26C	0.9800
C11—C12	1.4886 (15)	C27—C28	1.4952 (19)
C12—C17	1.3842 (17)	C27—H27A	0.9900
C12—C13	1.3886 (15)	C27—H27B	0.9900
C13—C14	1.3839 (17)	C28—H28A	0.9800
C13—H13	0.9500	C28—H28B	0.9800
C14—C15	1.373 (2)	C28—H28C	0.9800
C14—H14	0.9500		
			
C2—O1—C25	118.78 (9)	C17—C16—H16	120.0
C7—O2—C27	119.05 (10)	C16—C17—C12	120.17 (12)
C2—C1—C9	120.12 (10)	C16—C17—H17	119.9
C2—C1—C11	117.05 (10)	C12—C17—H17	119.9
C9—C1—C11	122.29 (10)	O4—C18—C19	121.56 (10)
O1—C2—C1	115.67 (10)	O4—C18—C8	118.61 (10)
O1—C2—C3	122.57 (10)	C19—C18—C8	119.83 (9)
C1—C2—C3	121.66 (11)	C20—C19—C24	119.00 (11)
C4—C3—C2	119.15 (11)	C20—C19—C18	122.01 (10)
C4—C3—H3	120.4	C24—C19—C18	118.96 (10)
C2—C3—H3	120.4	C21—C20—C19	120.60 (11)
C3—C4—C10	121.70 (11)	C21—C20—H20	119.7
C3—C4—H4	119.1	C19—C20—H20	119.7
C10—C4—H4	119.1	C22—C21—C20	119.74 (12)
C6—C5—C10	121.79 (11)	C22—C21—H21	120.1
C6—C5—H5	119.1	C20—C21—H21	120.1
C10—C5—H5	119.1	C21—C22—C23	120.31 (12)
C5—C6—C7	119.08 (11)	C21—C22—H22	119.8
C5—C6—H6	120.5	C23—C22—H22	119.8
C7—C6—H6	120.5	C22—C23—C24	119.87 (12)
O2—C7—C8	115.47 (10)	C22—C23—H23	120.1
O2—C7—C6	122.95 (11)	C24—C23—H23	120.1
C8—C7—C6	121.56 (11)	C23—C24—C19	120.46 (12)
C7—C8—C9	120.10 (10)	C23—C24—H24	119.8
C7—C8—C18	116.25 (10)	C19—C24—H24	119.8
C9—C8—C18	123.15 (10)	O1—C25—C26	106.80 (10)
C8—C9—C1	124.53 (10)	O1—C25—H25A	110.4
C8—C9—C10	117.78 (10)	C26—C25—H25A	110.4
C1—C9—C10	117.69 (10)	O1—C25—H25B	110.4
C5—C10—C4	120.66 (11)	C26—C25—H25B	110.4
C5—C10—C9	119.65 (11)	H25A—C25—H25B	108.6
C4—C10—C9	119.69 (11)	C25—C26—H26A	109.5
O3—C11—C12	121.04 (10)	C25—C26—H26B	109.5
O3—C11—C1	118.37 (9)	H26A—C26—H26B	109.5
C12—C11—C1	120.57 (9)	C25—C26—H26C	109.5
C17—C12—C13	119.30 (11)	H26A—C26—H26C	109.5
C17—C12—C11	118.99 (10)	H26B—C26—H26C	109.5
C13—C12—C11	121.71 (10)	O2—C27—C28	107.10 (11)
C14—C13—C12	120.42 (12)	O2—C27—H27A	110.3
C14—C13—H13	119.8	C28—C27—H27A	110.3
C12—C13—H13	119.8	O2—C27—H27B	110.3
C15—C14—C13	119.82 (12)	C28—C27—H27B	110.3
C15—C14—H14	120.1	H27A—C27—H27B	108.5
C13—C14—H14	120.1	C27—C28—H28A	109.5
C14—C15—C16	120.36 (12)	C27—C28—H28B	109.5
C14—C15—H15	119.8	H28A—C28—H28B	109.5
C16—C15—H15	119.8	C27—C28—H28C	109.5
C15—C16—C17	119.93 (13)	H28A—C28—H28C	109.5
C15—C16—H16	120.0	H28B—C28—H28C	109.5
			
C25—O1—C2—C1	−161.30 (10)	C2—C1—C11—O3	−108.29 (12)
C25—O1—C2—C3	22.47 (15)	C9—C1—C11—O3	63.28 (14)
C9—C1—C2—O1	−176.52 (9)	C2—C1—C11—C12	70.21 (13)
C11—C1—C2—O1	−4.75 (14)	C9—C1—C11—C12	−118.21 (11)
C9—C1—C2—C3	−0.25 (16)	O3—C11—C12—C17	1.58 (17)
C11—C1—C2—C3	171.51 (10)	C1—C11—C12—C17	−176.88 (11)
O1—C2—C3—C4	175.92 (10)	O3—C11—C12—C13	−178.72 (11)
C1—C2—C3—C4	−0.08 (16)	C1—C11—C12—C13	2.81 (16)
C2—C3—C4—C10	0.64 (17)	C17—C12—C13—C14	0.74 (19)
C10—C5—C6—C7	0.25 (18)	C11—C12—C13—C14	−178.95 (11)
C27—O2—C7—C8	164.97 (11)	C12—C13—C14—C15	0.0 (2)
C27—O2—C7—C6	−13.53 (17)	C13—C14—C15—C16	−0.6 (2)
C5—C6—C7—O2	176.45 (11)	C14—C15—C16—C17	0.4 (2)
C5—C6—C7—C8	−1.97 (18)	C15—C16—C17—C12	0.3 (2)
O2—C7—C8—C9	−176.28 (9)	C13—C12—C17—C16	−0.9 (2)
C6—C7—C8—C9	2.25 (17)	C11—C12—C17—C16	178.82 (13)
O2—C7—C8—C18	−4.12 (14)	C7—C8—C18—O4	−105.71 (12)
C6—C7—C8—C18	174.41 (10)	C9—C8—C18—O4	66.19 (14)
C7—C8—C9—C1	178.17 (10)	C7—C8—C18—C19	73.69 (13)
C18—C8—C9—C1	6.58 (16)	C9—C8—C18—C19	−114.42 (12)
C7—C8—C9—C10	−0.83 (15)	O4—C18—C19—C20	−176.90 (11)
C18—C8—C9—C10	−172.42 (9)	C8—C18—C19—C20	3.72 (16)
C2—C1—C9—C8	−178.97 (10)	O4—C18—C19—C24	1.44 (17)
C11—C1—C9—C8	9.71 (16)	C8—C18—C19—C24	−177.94 (11)
C2—C1—C9—C10	0.03 (14)	C24—C19—C20—C21	0.84 (18)
C11—C1—C9—C10	−171.29 (9)	C18—C19—C20—C21	179.18 (11)
C6—C5—C10—C4	−179.28 (11)	C19—C20—C21—C22	−0.3 (2)
C6—C5—C10—C9	1.12 (17)	C20—C21—C22—C23	−0.6 (2)
C3—C4—C10—C5	179.54 (11)	C21—C22—C23—C24	0.9 (2)
C3—C4—C10—C9	−0.86 (17)	C22—C23—C24—C19	−0.3 (2)
C8—C9—C10—C5	−0.82 (15)	C20—C19—C24—C23	−0.53 (19)
C1—C9—C10—C5	−179.89 (10)	C18—C19—C24—C23	−178.92 (12)
C8—C9—C10—C4	179.57 (10)	C2—O1—C25—C26	165.89 (11)
C1—C9—C10—C4	0.50 (15)	C7—O2—C27—C28	−171.15 (11)

### Hydrogen-bond geometry (Å, º)

**Table:**

*D*—H···*A*	*D*—H	H···*A*	*D*···*A*	*D*—H···*A*
C14—H14···O3^i^	0.95	2.37	3.2404 (16)	153
C21—H21···O4^ii^	0.95	2.39	3.3326 (16)	171

Symmetry codes: (i) *x*+1, *y*, *z*; (ii) *x*−1, *y*, *z*.

## References

[bb1] Burla, M. C., Caliandro, R., Camalli, M., Carrozzini, B., Cascarano, G. L., De Caro, L., Giacovazzo, C., Polidori, G., Siliqi, D. & Spagna, R. (2007). *J. Appl. Cryst.* **40**, 609–613.

[bb2] Burnett, M. N. & &Johnson, C. K. (1996). *ORTEPIII* Report ORNL-6895. Oak Ridge National Laboratory. Tennessee, USA.

[bb3] Higashi, T. (1999). *NUMABS* Rigaku Corporation, Tokyo, Japan.

[bb4] Muto, T., Sasagawa, K., Okamoto, A., Oike, H. & Yonezawa, N. (2012). *Acta Cryst.* E**68**, o1200. 10.1107/S1600536812012202 PMC3344137 22606140

[bb5] Nakaema, K., Watanabe, S., Okamoto, A., Noguchi, K. & Yonezawa, N. (2008). *Acta Cryst.* E**64**, o807. 10.1107/S1600536808007009 PMC2961268 21202298

[bb6] Nishijima, T., Kataoka, K., Nagasawa, A., Okamoto, A. & Yonezawa, N. (2010). *Acta Cryst.* E**66**, o2904–o2905. 10.1107/S1600536810041346 PMC3009325 21589080

[bb7] Okamoto, A., Mitsui, R. & Yonezawa, N. (2011). *Chem. Lett.* **40**, 1283–1284.

[bb8] Okamoto, A. & Yonezawa, N. (2009). *Chem. Lett.* **38**, 914–915.

[bb9] Rigaku (1998). *PROCESS-AUTO* Rigaku Corporation, Tokyo, Japan.

[bb10] Sasagawa, K., Muto, T., Okamoto, A., Oike, H. & Yonezawa, N. (2011). *Acta Cryst.* E**67**, o3354. 10.1107/S1600536811048550 PMC3238999 22199848

[bb11] Sheldrick, G. M. (2008). *Acta Cryst.* A**64**, 112–122. 10.1107/S0108767307043930 18156677

[bb12] Tsumuki, T., Hijikata, D., Okamoto, A., Oike, H. & Yonezawa, N. (2011). *Acta Cryst.* E**67**, o2095. 10.1107/S1600536811028054 PMC3213537 22091114

